# Supplementary material for: Sustainable Dengue Prevention and Management: Integrating Dengue Vaccination Strategies with Population Perspectives
Source: Vaccines (Basel). 2024 Feb 12;12(2):184. doi: 10.3390/vaccines12020184 (PMC10892244; doi:10.3390/vaccines12020184)
Supplement: Supplementary file 1 [file vaccines-12-00184-s001.zip › vaccines-2818763-supplementary.pdf]

Supplementary Material 1

DISCUSSION GUIDE FOR INTERVIEW

INTRODUCTION

Today's objective is to understand health system readiness in implementing a dengue prevention and management program that truly incorporates population views and perspectives.

Please consider your country, regional and global experience where appropriate

Dengue prevention and management program here refers to THE 3-PRONGED APPROACH - dengue vaccination, vector control and education. The education subcomponent considers the domains of dengue viruses and disease, vector control measures, and dengue vaccination

CFIR BRIEFING

We'll be using the CFIR framework today to guide our discussion.

Looking at the showcard, we'll be looking at how the different variables potentially facilitate or obstruct the successful rollout of the intervention - THE 3-PRONGED APPROACH

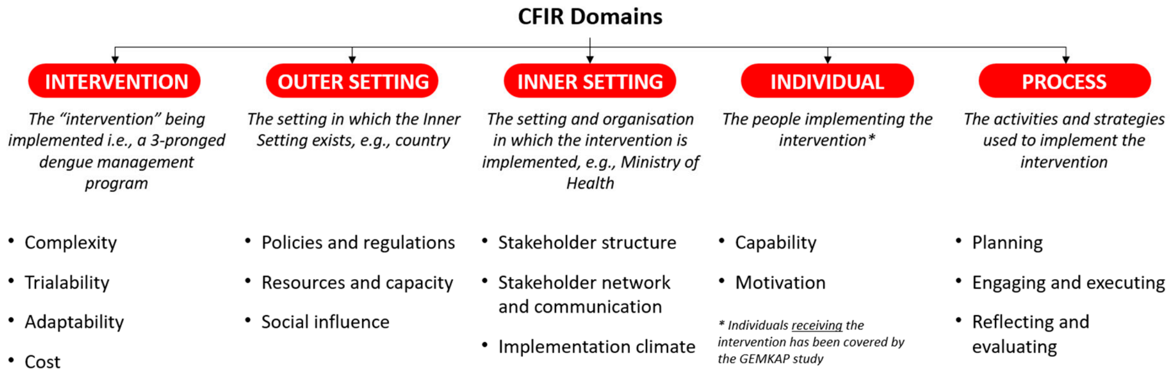

OVERVIEW

Thinking now about the 3-PRONGED APPROACH to dengue prevention and management - vaccine, vector control and education

1. Which of these CFIR LAYERS AND VARIABLES would you say are critical - either as a barrier to address or a facilitator to progress

INTERVENTION

Now thinking specifically about the 3-PRONGED APPROACH

2. To what extent do countries typically understand their population's views regarding vaccine implementation programs?
    a. How can this be improved?

3. How have health systems successfully integrated population views in health/ vaccine management programs in the past?
    a. Best practices
    b. Drivers, facilitators
    c. Barriers

4. Incorporating population views in a disease management program is undoubtedly complex, especially if the approach involves 3, separate but NOT mutually exclusive platforms - vaccination, vector control and education
    a. How have complex programs been successfully managed in the past?
    b. What are the drivers of this complexity?

OUTER SETTING

Government policies and regulations shape country practices and can have a large impact on health practices and the success of health programs.

5. Thinking of your experience either locally, regionally, or globally, what examples of government support in terms of policies, regulations and resources have led to successful outcomes in health or vaccination programs?

    a. FUNDING
        i. Cost and affordability
        ii. Incentives
    b. INFRASTRUCTURE
        i. Healthcare facilities
            1. Vaccination clinics
            2. Diagnostic labs
            3. Treatment wards
        ii. Vector control infrastructure
        iii. Educational infrastructure
        iv. Communication and social mobilization infrastructure
    c. COERCION / FINES
    d. Others

6. One of the GEMKAP survey findings was that a significant, albeit minority segment of the population did not see the need for a 3-pronged approach towards dengue prevention and management and believed that it was necessary to only implement one or two of those approaches at any one time
    a. Given the sizeable chunk of this population view and the corresponding social influence/pressure/expectations – what would we need to think about to address this potential resistance?

7. Disease management programs are often deprioritized relative to other more urgent national concerns. Based on your experience
    a. How, if at all, can a dengue prevention and management program be prioritized for attention and seen as a government priority?

INNER SETTING

Thinking now about the inner setting:

8. Which are the key organizations or bodies involved in rolling out such a program?
    a. What are their respective roles and responsibilities?
    b. To what extent is collaboration and coordination necessary?
       Prompt: e.g., is there a central stakeholder that should lead and spearhead the implementation?

9. How effectively have these organizations collaborated/coordinated in the past? How can this be optimized?
    a. Best practices
    b. Drivers, facilitators
    c. Barriers

INDIVIDUALS

10. Results from the GEMKAP study indicated that these stakeholders - HCPs, religious leaders, community leaders, influencers, etc. should actively work with the government when implementing a dengue prevention and management program - what has been the experience in the past in bringing these stakeholders together?
    a. Any other important stakeholders to engage?

11. How should/can these stakeholders be galvanized and coordinated?
    a. Knowledge levels, perceived need for change
    b. Sense of urgency
    c. What needs to be done to get these stakeholders ready for implementation?

12. Training for on the ground implementors has been identified as an important variable in the rollout of any disease management program
    a. True, false?
    b. If true, what type of training why

PROCESS

A successful dengue prevention and management program once implemented, would require consistent execution, periodic assessments and adapting in the face of changing circumstances and populations views/perspectives.

13. How do we win BOTH hearts and minds?

14. How can a dengue prevention and management program adapt to changes over time?

15. Examples of vaccine implementation programs that have been able to flex and adapt to specific needs of sub-populations
    a. Types of sub-populations to consider

16. What are some best practices that allow a dengue prevention and management program to be scalable and sustainable on the national level? Especially given the complexity of the 3-pronged approach

CASE STUDIES

17. Thinking back to your experience – either locally, regionally, or globally. Can you provide examples of when a disease management program was successfully implemented? What were the key ingredients leading to this success?

18. How was success determined?
    a. Metrics and KPIs

19. Reviewing the CFIR framework layers and variables
    a. Layer

        i. Which has an overweight role in determining the success of a dengue prevention and management program rollout?
        ii. Which layer is least important?
        iii. Why?
    b. Variable
        i. Most important?
        ii. Last important?
        iii. Why?

20. How about examples of when a disease management program was a failure. What caused this failure?
